# Myocardial viability as integral part of the diagnostic and therapeutic approach to ischemic heart failure

**DOI:** 10.1007/s12350-015-0096-5

**Published:** 2015-03-03

**Authors:** Jeroen J. Bax, Victoria Delgado

**Affiliations:** Department of Cardiology, Leiden University Medical Center, Albinusdreef 2, 2300 RC Leiden, The Netherlands

**Keywords:** Ischemic heart failure, myocardial viability, echocardiography, nuclear imaging, magnetic resonance, computed tomography

## Abstract

Chronic heart failure is a major public-health problem with a high prevalence, complex treatment, and high mortality. A careful and comprehensive analysis is needed to provide optimal (and personalized) therapy to heart failure patients. The main 4 non-invasive imaging techniques (echocardiography, magnetic resonance imaging, multi-detector-computed tomography, and nuclear imaging) provide information on cardiovascular anatomy and function, which form the basis of the assessment of the pathophysiology underlying heart failure. The selection of imaging modalities depends on the information that is needed for the clinical management of the patients: (1) underlying etiology (ischemic vs non-ischemic); (2) in ischemic patients, need for revascularization should be evaluated (myocardial ischemia/viability?); (3) left ventricular function and shape assessment; (4) presence of significant secondary mitral regurgitation; (5) device therapy with cardiac resynchronization therapy and/or implantable cardiac defibrillator (risk of sudden cardiac death). This review is dedicated to assessment of myocardial viability, however “isolated assessment of myocardial viability” may be clinically not meaningful and should be considered among all those different variables. This complete information will enable personalized treatment of the patient with ischemic heart failure.

**Electronic supplementary material:**

The online version of this article (doi:10.1007/s12350-015-0096-5) contains supplementary material, which is available to authorized users.

## Introduction

Chronic heart failure has become one of the clinically most important diseases of the heart. The prevalence is high; the treatment is complex, and the mortality is significant. The most detailed report comes from the update of heart disease and stroke statistics from the American Heart Association^1^-^3^ and is discussed in detail in the most recent American Heart Association/American College of Cardiology guidelines.^4^

Importantly, 5.1 million patients in the United States have heart failure, with a rising prevalence. Moreover, it is indicated that, for the American population, the lifetime risk of developing heart failure is 20% for individuals of age 40 years or more.^5^ Per year, more than 650,000 new patients are diagnosed with heart failure, and the incidence increases with increasing age, with more than 80 patients per 1000 individuals older than 85 years suffering of heart failure. Per year, more than 1 million patients are hospitalized with heart failure as the primary diagnosis, with a 1-month re-hospitalization rate of 25%.^1^ The costs for the care of patients with heart failure is high, exceeding $40 billion per year, with more than 50% of costs allocated to hospitalizations.^1^ Finally, the mortality is high, with approximately 50% of patients surviving 5 years after being diagnosed with heart failure.^1^ Specifically, in the Atherosclerosis Risk in Communities study, the 1- and 5-year mortality was 10% and 42%, respectively.^6^,^7^

It has been pointed out that a careful and comprehensive analysis is needed to provide optimal (and personalized) therapy to these patients.^4^,^8^,^9^ Most of the information can be provided by non-invasive imaging. Currently, the main 4 non-invasive imaging techniques include echocardiography, magnetic resonance imaging (MRI), multi-detector-computed tomography (MDCT), and nuclear imaging (positron emission tomography [PET] and single-photon emission-computed tomography [SPECT]). These imaging techniques can provide information on cardiovascular anatomy and function, which form the basis of the assessment of the pathophysiology underlying heart failure. The selection of imaging modalities depends on the information that is needed for the clinical management of the individual patient. In addition, the choice depends not only on local availability, but also local expertise and experience with the modalities and techniques.

**Figure 1:**
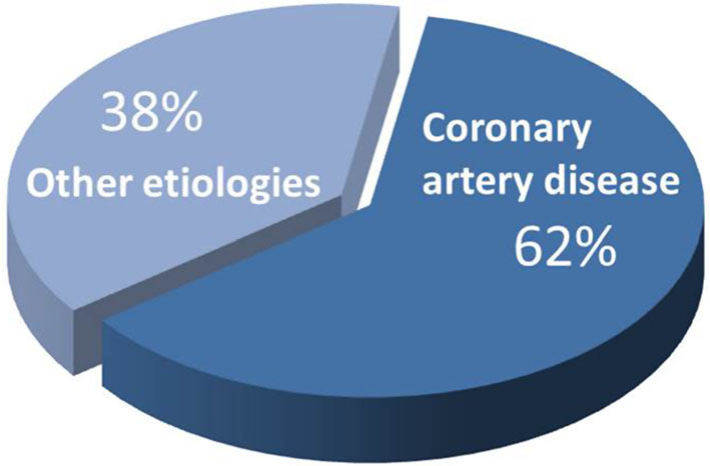
Heart failure etiology. From 24 multicenter heart failure trials, including 43,568 heart failure patients, 62% of patients had an ischemic etiology

The key information that is needed for comprehensive evaluation (for diagnosis and to determine therapeutic options) is summarized in Table 1. The most important issue is the etiology underlying heart failure: this differentiates between ischemic and non-ischemic cardiomyopathy, which is crucial for therapeutic options. Most patients will have ischemic cardiomyopathy; Gheorghiade et al^10^ summarized the data from 24 trials published in the New England Journal of Medicine during the period of 1986-2005, focusing on medical therapies in heart failure (Figure 1). These trials included 43,568 patients, and the prevalence of coronary artery disease was 62%; this percentage is probably an underestimation, since a significant percentage of patients did not undergo coronary angiography. Invasive coronary angiography remains the modality of choice to visualize the coronary arteries and detect or exclude coronary atherosclerosis/stenosis. However, non-invasive imaging modalities (MRI, MDCT) can also visualize the coronary arteries and detect coronary stenoses. MRI has been used extensively for coronary artery imaging. Particularly MDCT coronary angiography has been increasingly used for non-invasive angiography with a very high diagnostic accuracy: a recent meta-analysis with 89 studies and 7,516 patients showed a sensitivity of 97.2% and a specificity of 87.4%.^11^ MDCT coronary angiography has also been used successfully in patients with heart failure of unknown etiology,^12^ but it should be stressed that many patients with heart failure have elevated heart rate, whereas for MDCT coronary angiography low heart rates (<60 beats per minute) are needed.

**Table 1:** Information needed in patients with heart failure

Etiology: ischemic or non-ischemic heart failure?
Revascularization needed: is there ischemia and/or viability?
LV function, LV size and LV shape (LV aneurysm?)
Mitral valve: is there severe secondary mitral regurgitation?
Is ICD and/or CRT needed?

*CRT*, cardiac resynchronization therapy; *ICD*, implantable cardiac defibrillator; *LV*, left ventricular

In patients with ischemic cardiomyopathy, the need for revascularization needs to be considered and assessment of ischemia and viability is required. Next, the function, the size and the shape of the left ventricle need to be assessed: function is important for prognosis and potential indication for device therapy, whereas assessment of shape is important to evaluate the presence of severe left ventricular (LV) aneurysms. Also, patients with severe heart failure often have secondary mitral regurgitation: the prevalence of moderate or severe secondary mitral regurgitation in heart failure patients ranges between 24% and 30% and is associated with poor prognosis.^13^,^14^ If severe mitral regurgitation is present, mitral valve repair should be considered. Finally, patients with severe heart failure should be considered for device therapy, i.e., implantable cardiac defibrillator (ICD) device with or without cardiac resynchronization therapy (CRT). The need for these devices is based mainly on the LV ejection fraction (EF) and the duration of the QRS complex on the electrocardiogram (ECG), but imaging techniques may provide additional information that may be useful to improve patient selection.

This chapter is dedicated to assessment of myocardial viability, but the summary above already indicates that “isolated assessment of myocardial viability” is clinically not meaningful and should be considered among all the different variables that are summarized in Table 1. This complete information will enable personalized treatment of the patient with ischemic heart failure.

### Myocardial Viability—Pathophysiology and Definitions

Chronic contractile dysfunction can not only be related to scar tissue, but can also be related to hibernation or chronic stunning. In the presence of scar tissue, recovery of function will not occur, but in hibernation or chronic stunning, recovery of function may occur after revascularization. Viable myocardium is generally referred to as “alive myocardium,” independent of the contractile status of the myocardium. This however may not be preferred in the clinical setting, since the aim is to eventually predict improvement of function post-revascularization. Accordingly, the starting point in the discussion on viability should be the identification of regional dysfunction (usually assessed by echocardiography, but can also be detected by MRI or MDCT).

The distinction between hibernation and chronic stunning can be based on the assessment of myocardial blood flow; in hibernation resting blood flow is reduced, whereas in chronic stunning resting flow may still be preserved but flow reserve will be reduced.^15^ Clinically this differentiation may not always be feasible, but may also not be needed, since both entities require revascularization to improve in function and can be referred as viable, ischemically jeopardized myocardium.

**Figure 2:**
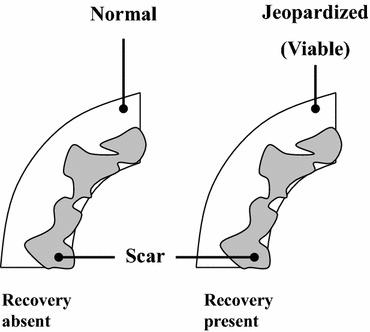
Prediction of functional recovery post-revascularization in dysfunctional segments with subendocardial scar is difficult. When the epicardial (non-infarcted) region is normal, no recovery will occur (*left panel*). However when the epicardial region contains jeopardized (viable) myocardium, the likelihood of recovery is high (*right panel*). Reproduced with permission from Kaandorp et al^16^

In contrast, the mixture of normal (non-jeopardized) myocardium and scar tissue (as may be encountered with subendocardial scar formation) will not improve in function and can be considered as viable, non-jeopardized myocardium (Figure 2).^16^

### Imaging Techniques to Detect Myocardial Viability—General Issues

A variety of non-invasive imaging techniques have been developed to detect viable myocardium in patients with ischemic heart failure. The different imaging techniques target different characteristics of viable myocardium (Table 2) and thus provide different information, which translate in different accuracies to predict improvement of function after revascularization.^17^

**Table 2:** Different characteristics of viable myocardium assessed with different imaging techniques

Imaging modality	Viability marker
Nuclear imaging	
SPECT using Tl-201	Perfusion, cell membrane integrity
SPECT using Tc-99m tracers	Perfusion, cell membrane integrity, intact mitochondria
PET (or SPECT) with FDG	Glucose utilization
Echocardiography	
Low-dose dobutamine infusion	Contractile reserve
MRI	
Low-dose dobutamine infusion	Contractile reserve
Intravenous contrast agents	Scar tissue

*FDG*, fluorine^18^-deoxyglucose; *MRI*, magnetic resonance imaging; *Tc-99m*, ^99^technetium; *Tl-201*, ^201^thallium

Most experience has been obtained with nuclear imaging using SPECT and PET; both techniques permit assessment of cardiac metabolism and perfusion. With SPECT, cell membrane or mitochondrial integrity can also be assessed.

Resting echocardiography or MRI can be used to assess LV end-diastolic wall thickness; it has been demonstrated that thinned myocardium (<6 mm) does not often improve in function after revascularization.^18^ Both echocardiography and MRI can be used in combination with low-dose dobutamine infusion to detect contractile reserve in dysfunctional myocardium. All these techniques aim at the detection of viability. It has been demonstrated that 40-50% of dysfunctional segments without contractile reserve may still have preserved perfusion and/or metabolism; some of these segments may still recover function after revascularization. It has been shown that loss of contractile reserve is associated with more severe ultrastructural damage and fibrosis formation and the severity of damage may eventually determine whether functional recovery is still possible.

In contrast to all the techniques discussed above which detect viable myocardium, contrast-enhanced MRI is the technique of choice, with the highest resolution, to detect scar tissue.

### The Different Imaging Techniques to Detect Viability

Most protocols and techniques have been described extensively over the last decades and have become widely implemented in the clinical setting.^17^

#### Positron emission tomography

Various PET tracers have been used (oxygen^15^-labeled water, carbon^11^-acetate, rubidium^82^), but for viability assessment, most experience has been obtained with metabolic imaging using fluorine^18^-deoxyglucose (FDG). This tracer is a glucose analog (1 OH-group is replaced by an F18 atom) and reflects cardiac glucose utilization. The first cellular uptake is comparable to glucose, and after phosphorylation to FDG-6-PO4, the tracer remains in the myocyte, which provides a strong signal for imaging. Since cardiac FDG uptake is strongly affected by metabolic circumstances, strict standardization of the metabolic milieu (plasma levels of glucose, free fatty acids, and insulin) is needed. To maximize cardiac glucose uptake, low free fatty acid levels with high glucose and insulin levels are required. This can be achieved with hyperinsulinemic euglycemic clamping, but this approach is time-consuming; alternative approaches include oral glucose loading and the use of nicotinic acid derivatives.

**Figure 3:**
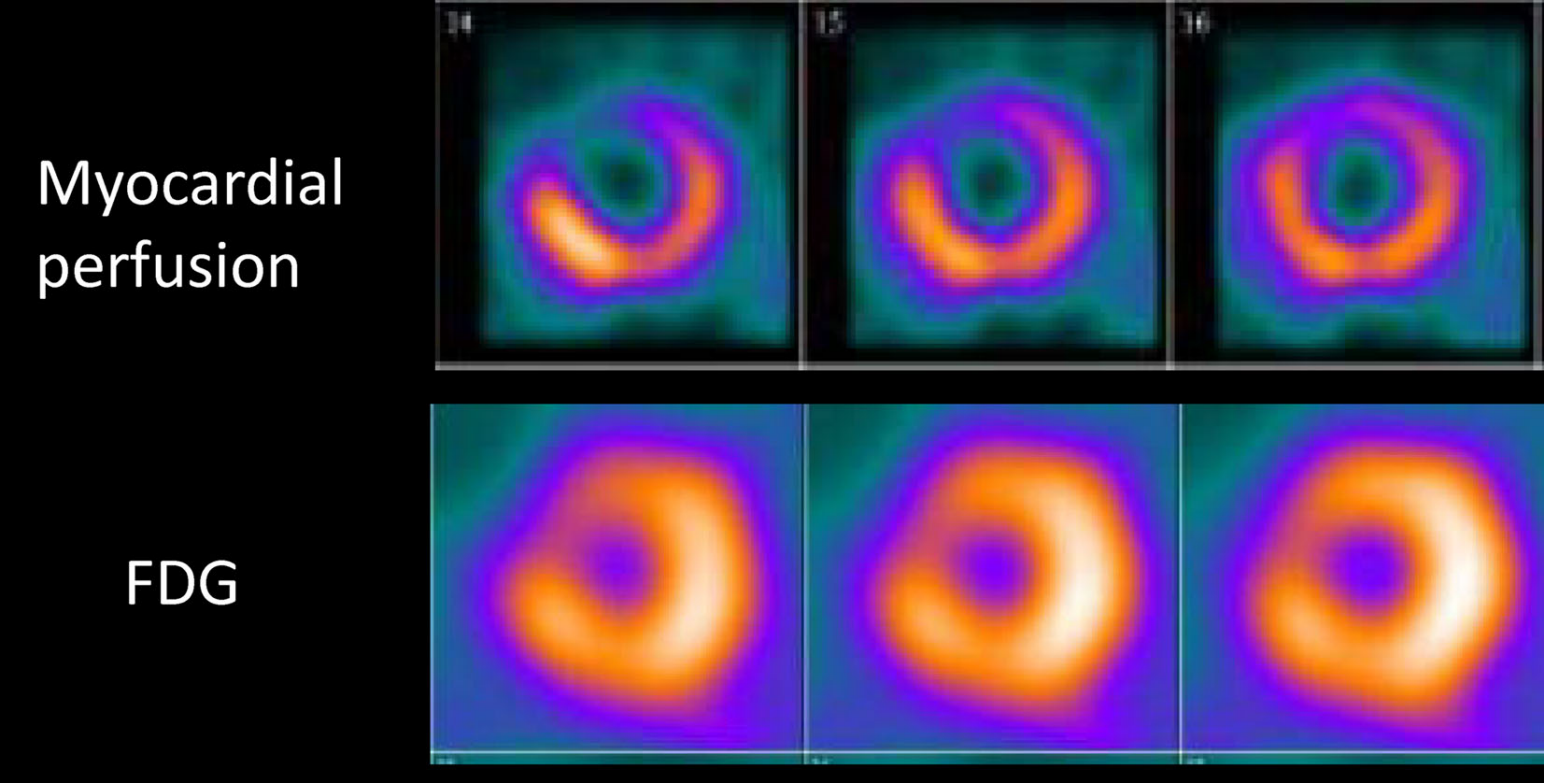
Example of a 76-year-old patient with previous anterior myocardial infarction and 2-vessel coronary artery disease on invasive coronary angiography (significant long lesion on the proximal left anterior descending coronary artery and dominant circumflex coronary artery with a significant lesion proximal). Left ventricular ejection fraction was 27%. Selected short-axis views of myocardial perfusion ^99^technetium tetrofosmin SPECT images show a perfusion defect in the anteroseptal wall. On fluorine^18^-deoxyglucose (FDG) SPECT images segments, uptake of radiopharmaceutical in the anteroseptal wall is visualized indicating perfusion-metabolic mismatch, pattern of myocardial viability

FDG imaging is combined with assessment of perfusion (usually nitrogen^13^-ammonia), and different patterns can be observed in areas of contractile dysfunction. Viable tissue may show normal perfusion and FDG uptake (which reflects chronic stunning) or reduced perfusion and preserved FDG uptake (referred to as mismatch pattern, which reflects hibernation). Scar tissue is characterized by reduced (non-transmural scar) or absent perfusion and FDG uptake (transmural scar). Of note, FDG imaging with SPECT and 511 keV collimators has also been developed successfully over the last decades (Figure 3).^19^

#### Single-photon emission-computed tomography

Both ^201^thallium chloride and ^99^technetium labelled SPECT tracers have been used. Following intravenous injection, the initial uptake of ^201^thallium predominantly reflects myocardial perfusion, and the prolonged retention/uptake reflects cell membrane integrity. Both stress-redistribution-reinjection and rest-redistribution protocols have been used in the clinical setting. Both protocols provide information on viability, whereas the first protocol includes also information on stress-inducible ischemia.

Various patterns of viability have been recognized in areas of contractile dysfunction, but they can be simplified as follows: any region with >50% tracer uptake on a resting image, and any defect with >10% increase in tracer uptake on the delayed images.^20^ It is however important to note that regions with >50% tracer uptake often do not improve in function, since these regions often contain a mixture of normal tissue and non-transmural scar.

The uptake and retention of the technetium-based tracers depend on perfusion, cell membrane integrity, and mitochondrial function. In areas of contractile dysfunction, a >50-60% tracer uptake on a resting image is frequently used as marker for viability. It has also been proposed to perform ^99^technetium sestamibi SPECT after nitrate administration, in order to increase blood flow (and tracer delivery) to severely hypoperfused areas.^20^ Typically, a resting image and a nitrate-enhanced image are acquired, and >10% increase in tracer uptake on the nitrate-enhanced images is considered indicative of viability.

#### Dobutamine stress echocardiography

During stepwise infusion of dobutamine, echocardiographic images are obtained evaluating regional wall motion. The initial phase includes low-dose dobutamine (5 to 10 mcg/kg/min) infusion, and the hall-mark of viability is “contractile reserve” which is improvement of contractile dysfunction during low-dose dobutamine infusion. The protocol can be extended to high-dose dobutamine infusion (up to 40 mcg/kg/min with addition of atropine), which allows detection of ischemia. The different wall motion responses during low–high dose dobutamine include (A) biphasic response (initial improvement followed by worsening of wall motion), (B) immediate worsening (direct deterioration of wall motion without initial improvement), (C) sustained improvement (improvement of wall motion without subsequent deterioration), and (D) no change (no change in wall motion at any stage). Pattern A represents viability with superimposed ischemia; pattern B represents severe ischemia due to critical coronary artery stenosis; pattern C relates to subendocardial infarction, and pattern D indicates transmural scar formation.

#### Magnetic resonance imaging

**Figure 4:**
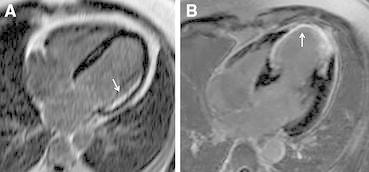
Contrast-enhanced magnetic resonance imaging for characterization of myocardial scar. Selected 4-chamber views on contrast-enhanced MRI of two patients with ischemic cardiomyopathy show non-transmural subendocardial scar (**A** *arrow*) and transmural scar (**B** *arrow*)

Areas with contractile dysfunction and a very thinned wall (<6 mm end-diastolic wall thickness) have a low likelihood of viability and functional recovery. Conversely, dysfunctional regions with preserved wall thickness do not always improve in function, and this is related to the presence of subendocardial scar (and the remainder of the wall being normal). Contractile reserve can be detected with MRI during dobutamine infusion as described above with echocardiography. An advantage of MRI is that wall thickening and motion can be quantitatively assessed, although in the clinical practice this rarely happens. The main contribution of MRI is the use of gadolinium-based contrast agents to detect scar tissue. Based on the excellent spatial resolution, contrast-enhanced MRI can differentiate between non-transmural and transmural scar tissue (Figure 4).

### Prediction of Functional Recovery After Revascularization

**Figure 5:**
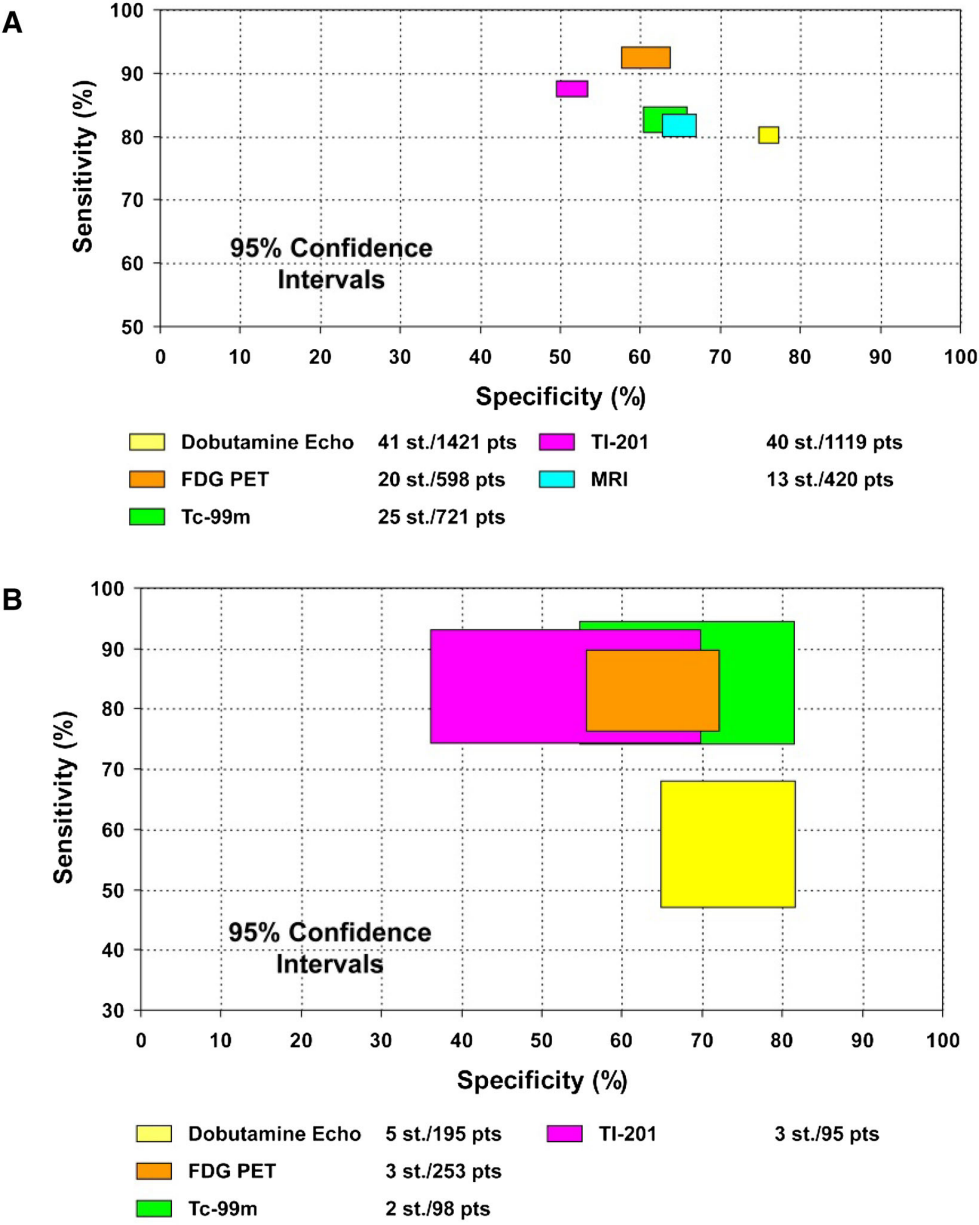
Comparison of sensitivities and specificities with 95% confidence intervals of the various techniques for the prediction of recovery of regional (**A**) and global (**B**) left ventricular function following coronary revascularization. Data based on Schinkel et al^17^. *Echo*, echocardiography; *FDG*, fluorine^18^-deoxyglucose; *MRI*, magnetic resonance imaging; *PET*, positron emission tomography; *Tc-99m*, ^99^technetium; *Tl-201*, ^201^thallium

A recent, very extensive report summarized the results of the viability studies focusing on prediction of functional recovery, and the results are summarized in Figure 5, and Tables 3 and 4.^17^

**Table 3:** Prediction of improvement of regional function after revascularization, comparison of different imaging techniques^17^

Technique	Sensitivity (%) (segments)	Specificity (%) (segments)	PPV (%) (segments)	NPV (%) (segments)
FDG PET (756 patients, 24 studies)	92 (1025/1111)	63 (620/984)	74 (1025/1389)	87 (620/716)
Tl-201 (1119 patients, 40 studies)	87 (2559/2931)	54 (1431/2655)	67 (2557/3810)	79 (1431/1814)
Tc-99m (721 patients, 25 studies)	83 (1170/1414)	65 (778/1189)	74 (1170/1581)	76 (778/1022)
DSE (1421 patients, 41 studies)	80 (3163/3941)	78 (3708/4746)	75 (3142/4174)	83 (3729/4518)
MRI wall thickness (100 patients, 3 studies)	95 (264/277)	41 (144/354)	56 (264/474)	92 (144/157)
MRI dobutamine (272 patients, 9 studies)	74 (564/766)	82 (720/878)	78 (564/722)	78 (720/923)
CE-MRI (178 patients, 5 studies)	84 (892/1060)	63 (610/964)	72 (892/1247)	78 (610/778)

*CE*, contrast-enhanced; *DSE*, dobutamine stress echocardiography; *FDG*, Fluorine^18^-deoxyglucose; *MRI*, magnetic resonance imaging; *NPV*, negative predictive value; *PPV*, positive predictive value; *Tc-99m*, ^99^technetium; *Tl-201*, ^201^thallium

**Table 4:** Prediction of improvement of global function after revascularization, comparison of different imaging techniques^17^

Technique	Sensitivity (%) (segments)	Specificity (%) (segments)	PPV (%) (segments)	NPV (%) (segments)
FDG PET (756 patients, 24 studies)	92 (1025/1111)	63 (620/984)	74 (1025/1389)	87 (620/716)
Tl-201 (1119 patients, 40 studies)	87 (2559/2931)	54 (1431/2655)	67 (2557/3810)	79 (1431/1814)
Tc-99m (721 patients, 25 studies)	83 (1170/1414)	65 (778/1189)	74 (1170/1581)	76 (778/1022)
DSE (1421 patients, 41 studies)	80 (3163/3941)	78 (3708/4746)	75 (3142/4174)	83 (3729/4518)
MRI wall thickness (100 patients, 3 studies)	95 (264/277)	41 (144/354)	56 (264/474)	92 (144/157)
MRI dobutamine (272 patients, 9 studies)	74 (564/766)	82 (720/878)	78 (564/722)	78 (720/923)
CE-MRI (178 patients, 5 studies)	84 (892/1060)	63 (610/964)	72 (892/1247)	78 (610/778)

*CE*, contrast-enhanced; *DSE*, dobutamine stress echocardiography; *FDG*, fluorine^18^-deoxyglucose; *MRI*, magnetic resonance imaging; *NPV*, negative predictive value; *PPV*, positive predictive value; *Tc-99m*, ^99^technetium; *Tl-201*, ^201^thallium

The highest sensitivity was obtained by FDG PET (92%), followed by SPECT with thallium-201 (87%) and technetium-99m-labeled agents (83%). The most specific approach was dobutamine stress echocardiography (specificity 78%). Improvement of global LV function (LVEF) is clinically more meaningful than improvement of segmental function. It is unclear how much viable myocardium is needed to result in the improvement of global LV function. Various studies have shown that recovery of global LV function may occur when at least 25% of the dysfunctional segments are viable.^21^-^27^ It should be emphasized however that various other factors also influence recovery of function (these are discussed below). Not only improvement of function is important, but also improvement in exercise capacity and heart failure symptoms are relevant, particularly from a patient’s perspective. This has been addressed in 8 studies,^22^,^23^,^28^-^33^ and the pooled analysis indicated that patients with significant viable tissue revealed an improvement in New York Heart Association (NYHA) functional class from 2.9 to 1.6 after revascularization, whereas the NYHA class did not change significantly in patients without viable tissue. In addition, 3 studies evaluated exercise capacity (expressed in METS) in relation to viability on FDG PET;^21^,^34^,^35^ in the viable patients, the exercise capacity improved from 4.4 to 5.7 after revascularization, whereas the change was minimal in non-viable patients (from 5.1 before to 5.9 after revascularization).

**Figure 6:**
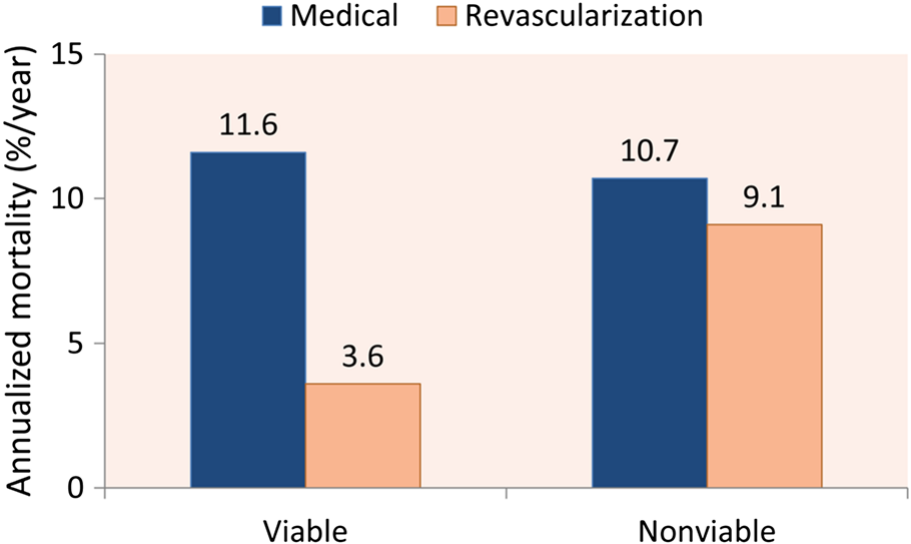
Annualized mortality rate of patients with and without significant viable myocardium according to treatment strategy. Results of a pooled analysis from 28 prognostic studies using different imaging techniques. Patients with viable myocardium who underwent coronary revascularization had the lowest mortality rate. Data based on Schinkel et al^17^

The most important issue is however the impact of viability on prognosis. A total of 28 retrospective viability studies have been reported including 3848 patients.^17^ The annualized mortality was lowest in the viable patients who underwent revascularization (3.6%), whereas annualized mortality was comparable in the other groups (ranging from 9.1% to 11.6%) (Figure 6). These retrospective studies suggested a direct relation between viability, improvement of function post-revascularization, translating in improved outcome. This topic was subsequently addressed in 1 prospective, randomized controlled trial: the STICH (Surgical Treatment for Ischemic Heart Failure) trial.^36^ In the larger part of the trial, 1212 patients with LVEF ≤ 35% and chronic coronary artery disease amenable to surgical revascularization were randomized to medical therapy (n = 602) or medical therapy and coronary artery bypass grafting surgery (CABG) (n = 610). In the medically treated group, death or hospitalization for cardiovascular events occurred in 411 patients (68%), as compared to 351 (58%) in the CABG group (hazard ratio with CABG, 0.74; 95% confidence interval, 0.64 to 0.85; *P* < 0.001). In the substudy, including 601 patients (298 medical therapy and CABG, 303 medical therapy alone), the relation between viability, therapy (surgery or medical therapy), and outcome was evaluated.^37^ The mortality was less in the viable patients (37%) as compared to the non-viable patients (51%, hazard ratio for death among patients with viable myocardium, 0.64; 95% confidence interval, 0.48 to 0.86; *P* = 0.003). Importantly, following correction for other baseline variables, this association with mortality was not significant (*P* = 0.21): these variables included LVEF, LV volumes, severity of symptoms, all indicators of more severe disease. In addition, there was no significant interaction between viability status and treatment regarding mortality (*P* = 0.53). Various issues could have affected the results of the STICH trial. First, as was pointed out in the beginning of this chapter, the focus should be on dysfunctional segments and in the STICH analysis all segments were included, and a large percentage of segments may be viable but have normal contractile function (and these cannot improve in function, and may thus affect the current results). This is also reflected in the distribution between viable and non-viable patients: 487 vs 114, while in the daily practice, the majority of patients with previous infarction does not contain viability in the infarcted area. In the current trial, the small cohort of non-viable patients is then randomized to 60 patients receiving medical therapy and 54 undergoing CABG. In addition, the viability techniques used were dobutamine stress echocardiography and ^99^technetium SPECT imaging, whereas FDG PET (the most sensitive viability assessment technique) and contrast-enhanced-MRI (the most accurate scar assessment technique) may have been preferred. Probably the most important issue is the beginning of this article: viability is only one part of the diagnostic and prognostic work-up of the patients with ischemic heart failure, and all other factors indicated in Table 1 will affect outcome. In the 2014 European Society of Cardiology guidelines on myocardial revascularization, it is recommended that myocardial revascularization should be considered in patients with chronic ischemic heart failure (LVEF ≤ 35%) in the presence of viable myocardium (class IIA, level of evidence B).^38^

### Other Information Needed in Chronic Ischemic Heart Failure: LV Function, Size, and Shape

**Figure 7:**
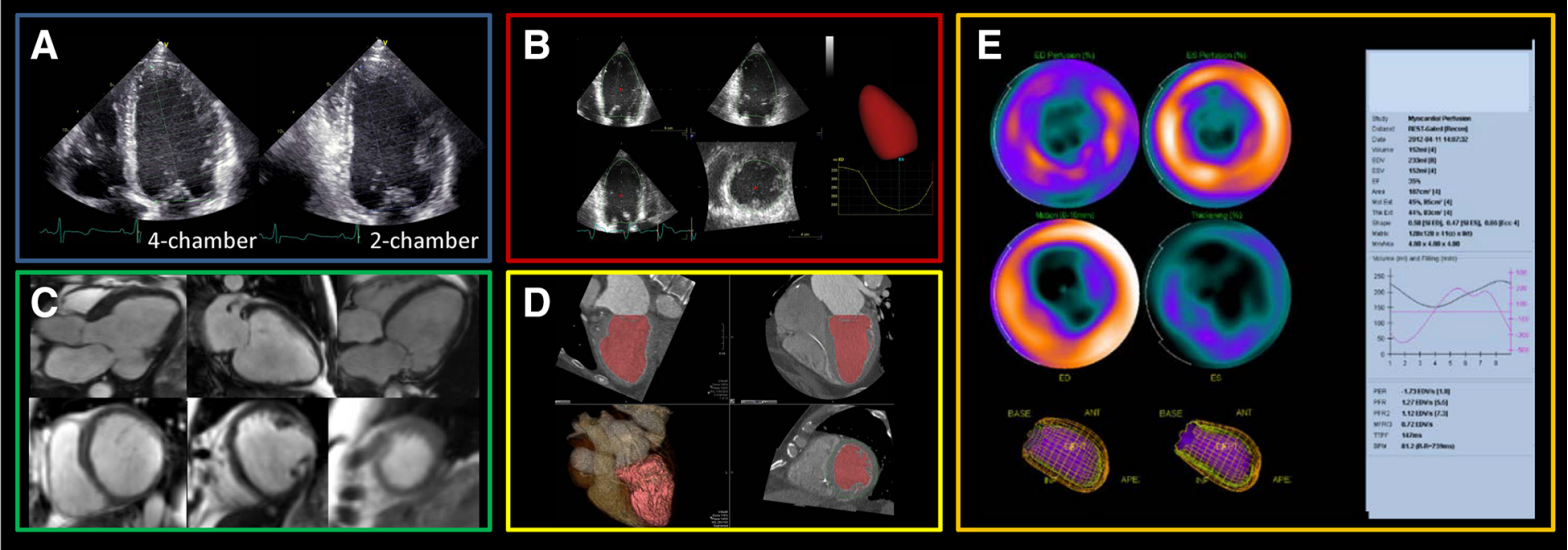
Assessment of left ventricular systolic function and geometry with current imaging modalities. **A** 2-dimensional transthoracic echocardiography; **B** 3-dimensional transthoracic echocardiography; **C** magnetic resonance imaging; **D** multi-detector row computed tomography; **E** ECG-gated single-photon emission-computed tomography

Left ventricular volumes and LVEF are important parameters for the risk stratification and management (particularly device therapy) of heart failure patients. The wide availability, accuracy, and safety make 2-dimensional transthoracic echocardiography the imaging technique of first choice (video 1).^9^ Besides LV dimensions, geometry, and LVEF, echocardiography provides information on diastolic function, valvular heart disease, pericardial disease, right ventricular function, and pulmonary systolic pressure. Internal LV linear dimensions can be measured from M-mode recordings from the parasternal long-axis view which is a reproducible and high temporal resolution method. However, in abnormal LV geometry, these measurements are not accurate, and assessment of LV volumes using the biplane disk summation method from the apical 2- and 4-chamber views is recommended (Figure 7).^39^ The addition of intravenous echocardiographic contrast is recommended when ≥2 contiguous LV endocardial segments are poorly visualized (video 1). These contrast agents enhance endocardial border definition and provide more reproducible measurements of volumes and LVEF, and compare well with MRI.^40^ Further improvement can be obtained by 3-dimensional echocardiography (Figure 7).^41^ Besides echocardiography, other 3-dimensional techniques such as MRI^42^ or ECG-gated MDCT provide high-quality measurements of LV volumes and LVEF (Figure 7, video 1).^43^ Finally, with ECG-gated myocardial perfusion SPECT accurate 3-dimensional measurements of LV volumes and EF can be obtained from the time volume curves (Figure 7).

**Figure 8:**
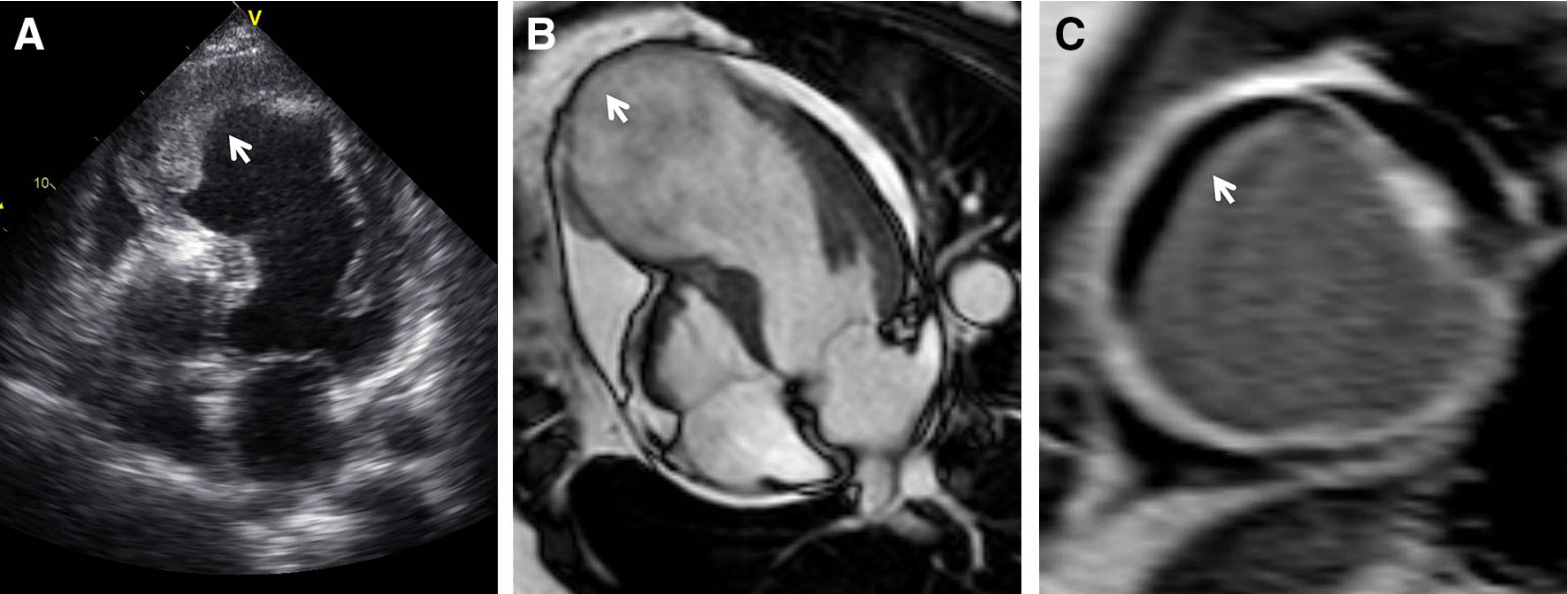
Example of a patient with large anterior myocardial infarction and subsequent formation of a large apical aneurysm. On transthoracic 2D echocardiography (**A**), the *arrow points* to the large apical aneurysm with thrombus formation. Magnetic resonance imaging shows a thin-walled apical aneurysm (*arrow*) extending from the mid septum to the apical lateral wall (**B**). On contrast-enhanced MRI (**C**), the short-axis view of the aneurysm shows hyperenhanced transmural scar (*white*) with a large apical thrombus (*black*, *arrow*)

In addition, LV shape is important, and particularly the formation of large LV aneurysms after infarction. Thrombus formation frequently occurs at these aneurysmatic sites, and surgical resection of dysfunctional myocardium and/or scar tissue can reduce LV size and restore normal geometry.^38^ Information on location and extent of LV aneurysms can be provided by echocardiography, MRI, MDCT, and gated SPECT (Figure 8, video 1).

### Additional Information: Severe Secondary Mitral Regurgitation

**Figure 9:**
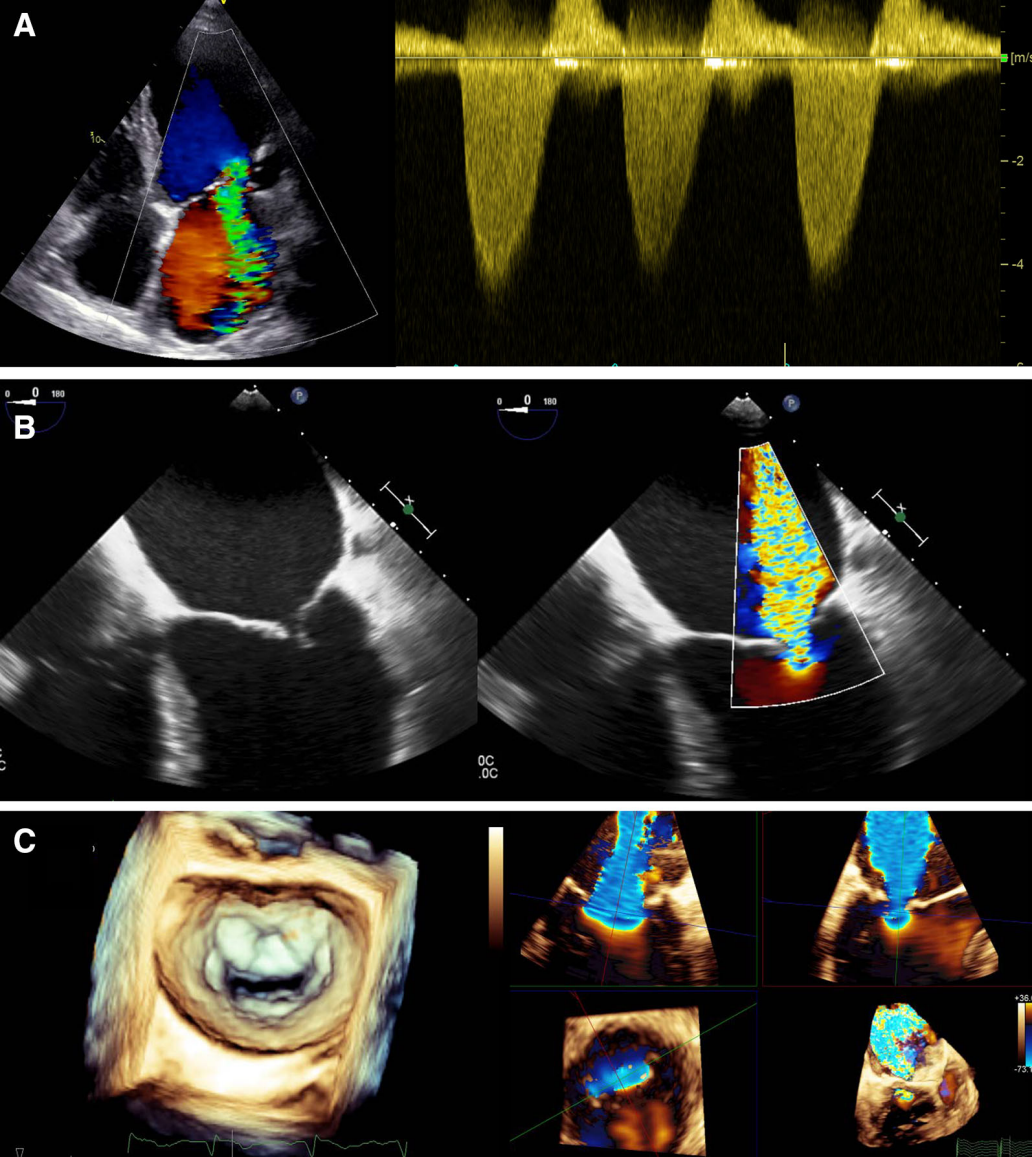
Assessment of secondary mitral regurgitation. Example of a 56-year-old patient with previous inferior myocardial infarction and chronically occluded right coronary artery who presented with dyspnea on exertion. On transthoracic color Doppler echocardiography, severe mitral regurgitation with an eccentric jet (*green*) adhering to the lateral left atrial wall and reaching the pulmonary veins (due to restriction of the posterior mitral leaflet) is shown (**A** *left*). Continuous wave Doppler shows a holosystolic dense signal of the regurgitant jet (**A** *right*). On transesophageal echocardiography, the lack of coaptation between the leaflets can be appreciated leading to large regurgitant jet on color Doppler view (**B**). On 3-dimensional transesophageal echocardiography, the en-face view of the mitral valve shows lack of coaptation at all the segments of the mitral leaflets (*left*) and the multiplanar reconstructions of the 3-dimensional color Doppler data show a large elongated effective regurgitant orifice (**C** *right*)

Secondary mitral regurgitation occurs frequently in patients with ischemic heart failure, and is characterized by a combination of reduced LV closing forces (due to LV dysfunction or dyssynchrony) and global and regional LV remodeling which leads to distortion of the subvalvular apparatus of the mitral valve, displacement of the papillary muscles, tethering of the mitral leaflets, and failure of mitral valve coaptation. Secondary mitral regurgitation results in LV volume overload, which further worsens LV remodeling and mitral valve incompetence. The presence of significant secondary mitral regurgitation provides incremental prognostic information over LVEF.^13^ In 1256 heart failure patients (60% ischemic etiology), significant secondary mitral regurgitation was associated with increased risk of heart failure hospitalization and all-cause mortality.^13^ Therefore, accurate quantification of secondary mitral regurgitation severity is crucial for clinical decision making in heart failure patients. Two-dimensional echocardiography remains the mainstay imaging technique to assess the severity and mechanism of secondary mitral regurgitation.^44^ Severe secondary mitral regurgitation is defined by an effective regurgitant orifice area ≥0.2cm^2^ and a regurgitant volume ≥30 mL/beat quantified using the proximal isovelocity surface area (PISA) method.^44^ While in central regurgitant jets, the PISA method is relatively accurate to quantify the severity of mitral regurgitation, in eccentric or multiple regurgitant jets or non-hemispheric regurgitant orifices, this method may be less accurate (Figure 9, video 2). In those cases, 3-dimensional imaging techniques may provide more accurate quantification of secondary mitral regurgitation severity (Figure 9, video 2).^45^ Furthermore, secondary mitral regurgitation is highly dynamic and strongly influenced by pressure and volume conditions. Therefore, in symptomatic heart failure patients with mild to moderate secondary mitral regurgitation, exercise echocardiography may help unmask severe mitral regurgitation. An increase in effective regurgitant orifice area ≥0.13 cm^2^ at peak stress has been associated with poor survival.^46^

The suitability for surgical restrictive mitral valve annulopasty is influenced by several geometrical aspects of the mitral valve and left ventricle, and also by the presence of extensive myocardial scar, which has been associated with increased mortality rates after surgical repair.^47^,^48^

### More Information: Sudden Cardiac Death

**Figure 10:**
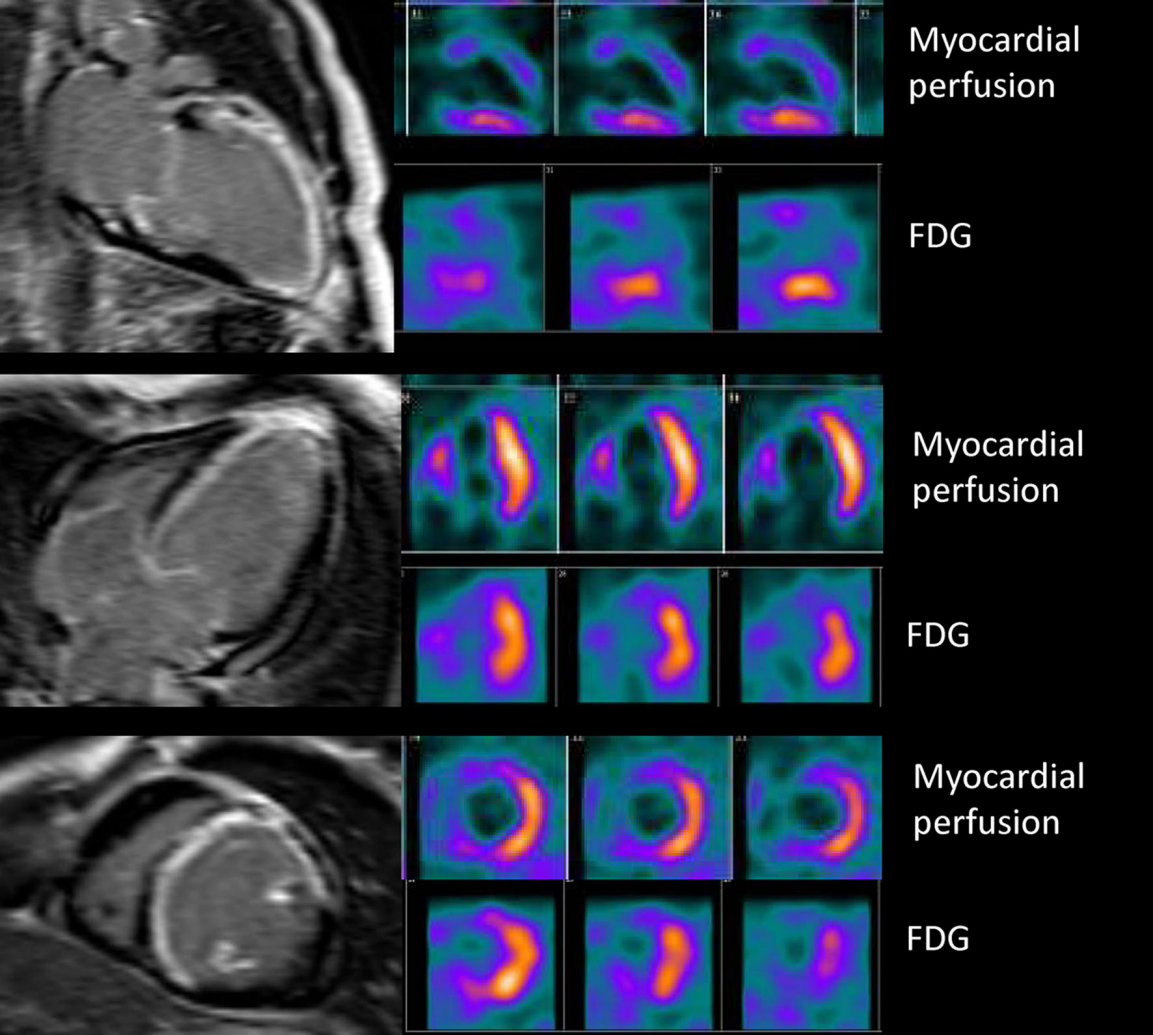
Myocardial scar and viability for assessment of risk of arrhythmic death. Example of a 57-year-old patient with anterior myocardial infarction and a left ventricular ejection fraction of 17%. Contrast-enhanced magnetic resonance images show large transmural scar in the anterior and septal walls and subendocardial (50% of the wall) in the inferoseptal wall. Selected vertical and horizontal long-axis and short-axis views of the left ventricle on ^99m^Technetium tetrofosmin SPECT show a perfusion defect of the septal, anterior and apical segments that match the defects on fluorine^18^-deoxyglucose SPECT. The patient received an implantable cardiac defibrillator (ICD) for primary prevention of sudden cardiac death. Two months later, the patient was admitted with an appropriate ICD shock

Patients with ischemic heart failure and depressed LVEF (≤35%) have an increased risk of arrhythmic death.^49^ Various trials have demonstrated the prophylactic benefit of an ICD in these patients.^50^-^53^ However, the percentage of patients that require ICD therapy (appropriate shocks) to prevent ventricular tachycardia/fibrillation (VT/VF) at follow-up is relatively low, suggesting that a substantial percentage of patients may not benefit from ICD.^54^ Currently, the LVEF (<30-35%) is used as the main selection criterium for ICD therapy.^55^ The precise substrate for VT/VF however is unknown, but may be related to scar tissue (infarct zone) and the heterogenous border zone around the infarct core (mixture of viable myocardium interspersed with fibrous and scar tissue). Non-invasive imaging may eventually help in patient selection for ICD implantation. The presence, extent, and characteristics of myocardial scar assessed with contrast-enhanced MRI have incremental prognostic value over LVEF to predict the occurrence of VT/VF.^56^ Subsequent studies quantifying the extent of contrast-enhanced tissue (scar mass) revealed that the arrhythmic risk increased significantly when the scar mass exceeded >1.4-5% of the LV volume (Figure 10).^57^,^58^ However, this arrhythmic risk reached a plateau at larger levels of scar mass suggesting that other factors contribute to the arrhythmogenic substrate, and the extent of the border zone has also been associated with increased risk of VT. Roes et al^59^ demonstrated that an extent of the border zone ≥16.7 g identified a group of patients with high incidence of appropriate ICD therapies. Each 10 g increase of border zone increased the risk of appropriate ICD therapies by 1.49 (95% confidence interval 1.01-2.2, *P* = 0.04) independently of LVEF and extent of infarct core.^59^

**Figure 11:**
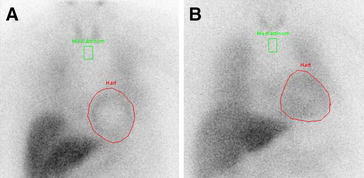
Cardiac innervation imaging with ^123^-iodine (^123^I)-metaiodobenzylguanidine (MIBG). The planar images show the global myocardial uptake (*red circles*) of ^123^I-MIBG of two patients with ischemic heart failure: patient A had a heart-to-mediastinum (H/M) ratio of 1.54, indicating low cardiac MIBG uptake, while patient B had an H/M ratio of 1.64, indicating more preserved MIBG uptake. Both patients received an implantable cardiac defibrillator (ICD). At follow-up, only patient A had appropriate ICD shocks while patient B remained free of ventricular arrhythmias

Possibly, another characteristic of the border zone is the presence of viable but (partially) denervated myocardium; the functional integrity of the sympathetic nerve terminals in this area can be evaluated with using radiolabeled norepinephrine analogs.^60^ This concept was recently addressed in the Prediction of ARrhythmic Events with Positron Emission Tomography trial.^61^ In 204 heart failure patients receiving an ICD, cardiac sympathetic innervation was quantified with ^11^C-meta-hydroxyephedrine (^11^C-HED) PET, perfusion was assessed with ^13^nitrogen-ammonia and viability was quantified with FDG. Patients with vs without ICD shocks had significantly larger areas of denervated myocardium (33 ± 10% vs 26 ± 11%, *P* < 0.001) and larger areas of denervated, viable myocardium (10 ± 6% vs 7 ± 5%, *P* = 0.02) despite having comparable LVEF. In addition, the extent of denervated myocardium was independently associated with ICD shocks (hazard ratio 1.069 per 1% increment, 95% confidence interval 1.023-1.117; *P* = 0.003).^61^ Similarly, ^123^-iodine (^123^I)-metaiodobenzylguanidine (MIBG) SPECT has been used to evaluate cardiac innervation. From the planar images, the heart-to-mediastinum (H/M) ratio and the washout rate quantify the global myocardial uptake of ^123^I-MIBG (Figure 11). The AdreView Myocardial Imaging for Risk Evaluation in Heart Failure trial, enrolling 961 heart failure patients (68% ischemic heart disease), showed that an H/M ratio <1.6 identified a cohort of patients with increased incidence of ventricular arrhythmias.^62^ The H/M ratio was an independent predictor of cardiac events (hazard ratio 0.36, 95% confidence interval 0.17-0.75; *P* = 0.006). In addition, from SPECT images, regional abnormalities in ^123^I-MIBG uptake have been associated with ventricular arrhythmias.^63^ Among 116 ICD recipients (74% ischemic heart failure), a late ^123^I-MIBG SPECT summed defect score >26 identified a subgroup of patients with high cumulative rates of ventricular arrhythmias at 3 years follow-up (52% vs 5%, *P* < 0.01).^63^ These studies suggest that imaging may potentially help in patient selection for ICD therapy.

### Final Information: Cardiac Resynchronization Therapy

Cardiac resynchronization therapy can also improve clinical outcomes of heart failure patients in NYHA functional class II-IV despite optimal medical therapy, with LVEF <35% and QRS duration >120 ms.^64^ The individual response varies significantly and imaging may help improve patient selection. Nuclear imaging techniques provide information on 2 important pathophysiological determinants of response to CRT: LV mechanical dyssynchrony^65^-^67^ and the extent and location of LV scar tissue.^66^,^68^,^69^

**Figure 12:**
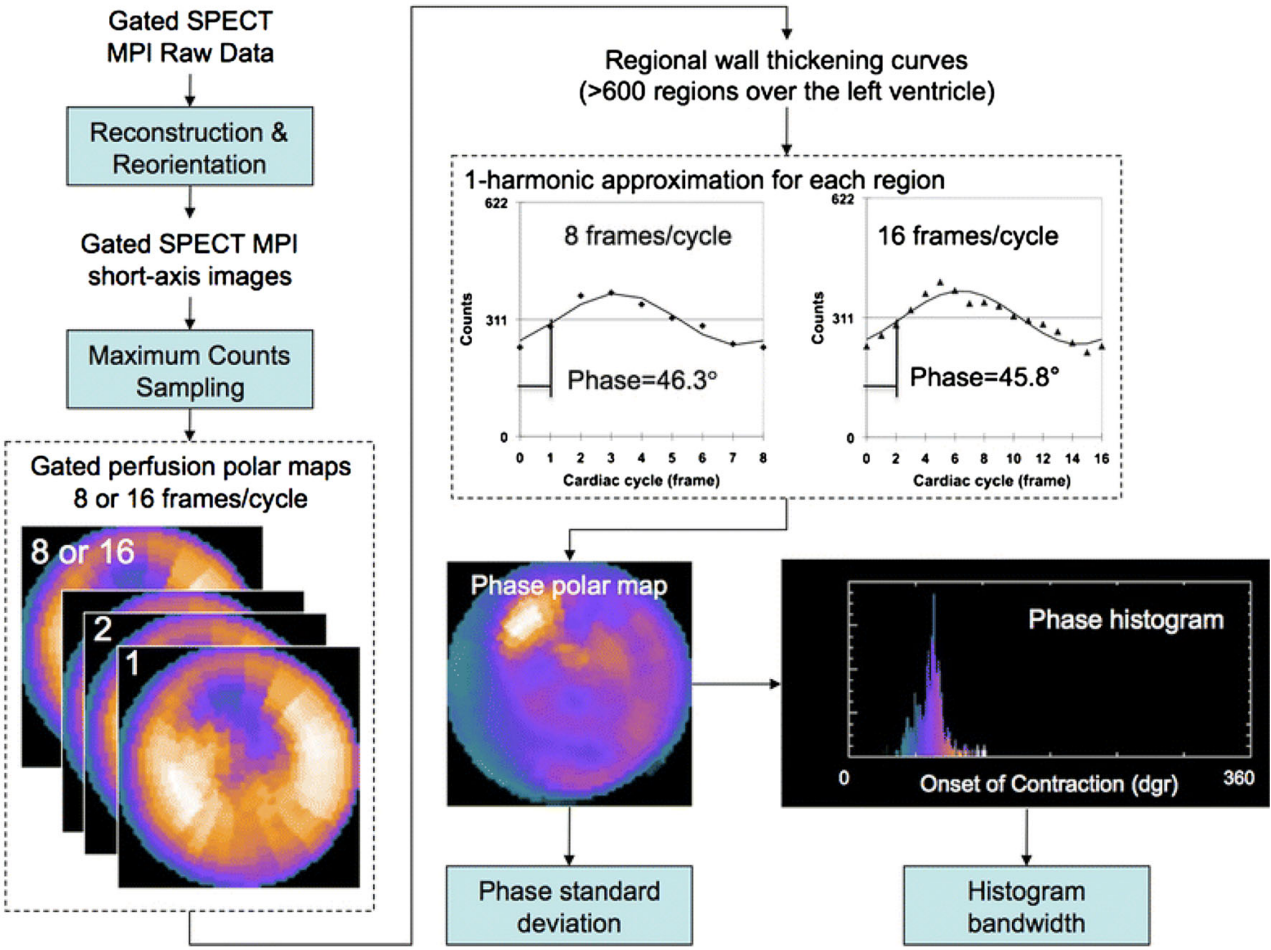
Phase analysis of ECG-gated SPECT myocardial perfusion imaging to assess LV dyssynchrony. From the reconstructed and reoriented ECG-gated SPECT myocardial perfusion imaging data, a gated short-axis image is obtained. On each temporal frame of the gated short-axis image, 3-dimensional sampling is performed to detect the regional maximum counts which represent regional wall thickening data. The first harmonic Fourier function is used to approximate the regional wall thickening data to calculate the phase angle for each region. From the regional phase angles, the phase distribution is derived and presented in a polar map or a histogram. Reproduced with permission from Chen et al^70^

Phase analysis measures the onset of mechanical contraction by approximating the variation of regional maximal counts over the cardiac cycle (which represents myocardial wall thickening) in SPECT myocardial perfusion imaging with Fourier harmonic functions (Figure 12).^70^ From the onset mechanical contraction phase distribution, the phase standard deviation and the phase histogram bandwidth are derived as quantitative indices of LV mechanical dyssynchrony. The relevance of these LV mechanical dyssynchrony indices to predict response to CRT was demonstrated in 40 patients with heart failure (70% ischemic cardiomyopathy).^65^ After 6 months of follow-up, 60% of patients revealed a symptomatic improvement (responders). Compared with non-responders, these patients had significantly larger histogram bandwidth (94 ± 23° vs 68 ± 21°, *P* < 0.01) and phase standard deviation (26 ± 6° vs 18 ± 5°, *P* < 0.01). Furthermore, accumulating evidence has shown that an LV lead placed at the latest activated region of the left ventricle is associated with good response to CRT.^71^ The phase polar maps of the left ventricle can be subdivided into 17 segments and the mean phase of each segment, which represents the timing of onset of mechanical contraction, can be displayed. The segment with the largest mean phase is identified as the latest activated segment. Furthermore, the presence of transmural scar at the segments targeted by the LV lead and the presence of significant scar burden have been associated with lack of response to CRT.^68^,^69^

**Figure 13:**
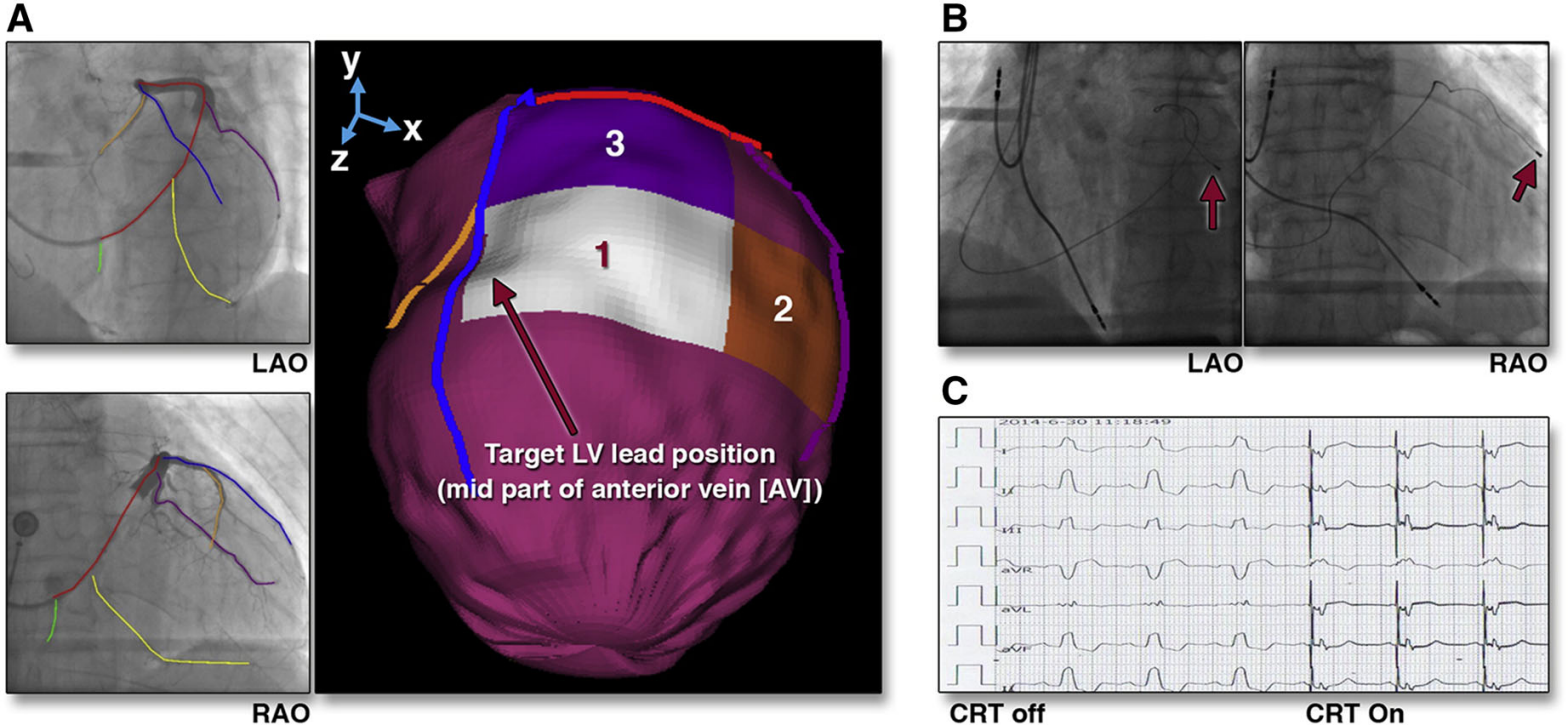
Fusion imaging of cardiac venous anatomy and left ventricular site of latest activation to guide left ventricular lead position. The SPECT-vein navigation tool kit permits fusion imaging of fluoroscopic venograms into a 3-dimensional LV epicardial surface extracted from ECG-gated SPECT myocardial perfusion imaging (**A**). The mid part of the anterior vein (AV, *blue line*) was aligned with the optimal site (white segment). **B** shows the final position of the LV lead on the left anterior oblique (LAO) and right anterior oblique (RAO) projections. **C** shows the post-implantation ECG with significant reduction of the QRS duration when CRT was activated (from 168 to 140 ms). Reproduced with permission from Zhou et al^72^

Eventually, the information on LV dyssynchrony, the site of latest activation, and scar tissue can be fused with fluoroscopic venograms into a 3-dimensional LV epicardial surface extracted from ECG-gated SPECT myocardial perfusion imaging (Figure 13).^72^ The clinical role of this tool needs to be demonstrated in prospective studies, but it highlights the potential use of nuclear imaging to select patients for CRT.

## Conclusion

The patient with ischemic heart failure needs careful, stepwise diagnostic analysis; non-invasive imaging can provide much of this information. Myocardial viability assessment is an important component, but should be integrated within all other relevant information. Once all the information is completed, a personalized therapeutic plan can be presented, making a personalized approach feasible to the individual patient.

## Case 1

A 59-year-old male presented at the outpatient clinic with heart failure symptoms since 3 weeks. The patient was a smoker and had hypertension treated with angiotensin converting enzyme inhibitors. On transthoracic echocardiography, a dilated left ventricle with a biplane left ventricular ejection fraction of 22% and global hypokinesia were observed (video 1). The invasive coronary angiography showed severe 3-vessel coronary artery disease (video 2). To assess the presence of viable myocardium, magnetic resonance imaging was requested. On cine acquisitions, the left ventricular end-diastolic and end-systolic volumes were 420 mL and 348 mL, respectively, and the left ventricular ejection fraction was 17% (video 3). The end-diastolic wall thickness was >6 mm. On contrast-enhanced magnetic resonance imaging, subendocardial scar <10% of the mid anterior and inferior walls was documented (Figure 1, arrows). In addition, low-dose dobutamine stress echocardiography was performed showing the significant increase in wall thickness and left ventricular function (video 4). Detection of ischemia with a higher dose of dobutamine was not performed. Based on the magnetic resonance and low-dose dobutamine stress echocardiography findings (end-diastolic wall thickness >6 mm and subendocardial scar of <10%, improvement of left ventricular systolic function during dobutamine) indicating the presence of myocardial viability, the patient was referred for surgical revascularization. Echocardiography at 1 year follow-up showed reduction of left ventricular volumes and improvement of left ventricular ejection fraction (video 5).

## Electronic supplementary material

Below is the link to the electronic supplementary material.

Video 1 Assessment of left ventricular systolic function. Panel A: 2-dimensional transthoracic echocardiography showing an apical 4-chamber view with reduced left ventricular ejection fraction. Panel B: Contrast-enhanced 3-dimensional triplane apical view of the left ventricle. Panel C: ECG-gated single-photon emission-computed tomography of the left ventricle showing apical dyskinesia. Panel D: assessment of left ventricular systolic function with multi-detector row computed tomography. Panel E: Cine magnetic resonance imaging showing the reconstructed 4-chamber view. The left ventricle is dilated, with reduced left ventricular ejection fraction and large thin-walled apical aneurysm (WMV 8802 kb)

Video 2 Evaluation of secondary mitral regurgitation. Panel A: 2-dimensional transthoracic color Doppler echocardiography showing the apical 4-chamber view and severe secondary mitral regurgitation with large eccentric jet along the lateral wall of the left atrium. Panel B: 2-dimensional transesophageal echocardiography showing the 4-chamber view focused on the mitral valve which shows restriction of the posterior mitral leaflet (arrow) and lack of coaptation. Panel C: 2-dimensional transesophageal color Doppler echocardiography showing the resultant severe secondary mitral regurgitation. Panel D: 3-dimensional full volume en-face view of the mitral valve showing a large area of lack of coaptation of the mitral leaflets. Panel E: On 3-dimensional transesophageal color Doppler echocardiography data, the multiplanar reformations can be oriented across the vena contracta of the regurgitant jet showing the elliptic shape of the regurgitant orifice (WMV 11693 kb)

Case 1. Figure 1 : Contrast-enhanced magnetic resonance imaging showing selected 2-chamber and short-axis views of the left ventricle. The dilated left ventricle revealed subendocardial scar (<10% transmurality) in the mid anterior and inferior walls (arrows) (TIFF 1256 kb)

Case 1. Video 1 2-dimensional transthoracic echocardiography showing the apical left ventricular 4-, 2- and 3-chamber views. The left ventricular ejection fraction was 22% (WMV 18966 kb)

Case 1. Video 2 Invasive coronary angiography showing severe stenosis of the proximal segments of the left anterior descending and circumflex coronary arteries (panel A) and chronically occluded right coronary artery (panel B) (WMV 12888 kb)

Case 1. Video 3 Cine magnetic resonance imaging showing the 4- and 2-chamber long-axis and the short-axis views of the left ventricle. The left ventricle is significantly dilated and shows global hypokinesia but no significant wall thinning (WMV 5990 kb)

Case 1. Video 4 Low-dose dobutamine stress echocardiography showing the apical 4-, 2- and 3-chamber views of the left ventricle. The left ventricular function and wall thickening improved at low-dose dobutamine (WMV 9670 kb)

Case 1. Video 5 After surgical revascularization, significant left ventricular reverse remodeling was observed with subsequent improvement in systolic function. Apical left ventricular 4-, 2- and 3-chamber views are shown (WMV 16591 kb)
