# Supplementary figures and images for: Myocardial viability as integral part of the diagnostic and therapeutic approach to ischemic heart failure
Source: J Nucl Cardiol. 2015 Mar 3;22(2):229–45. doi: 10.1007/s12350-015-0096-5 (PMC4490177; doi:10.1007/s12350-015-0096-5)

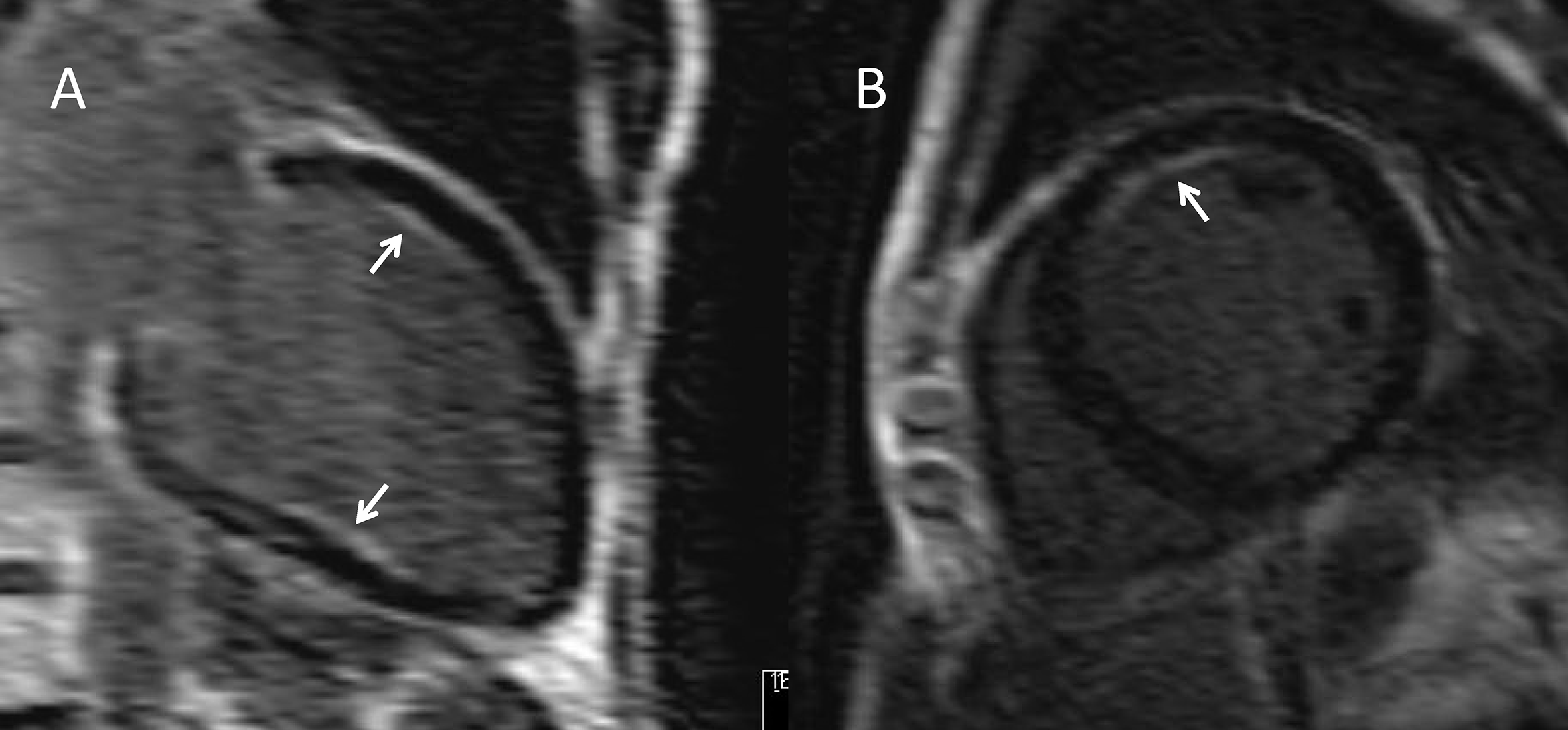

Supplement: Supplementary file 3 — : Contrast-enhanced magnetic resonance imaging showing selected 2-chamber and short-axis views of the left ventricle. The dilated left ventricle revealed subendocardial scar (<10% transmurality) in the mid anterior and inferior walls (arrows) (TIFF 1256 kb) [file 12350_2015_96_MOESM3_ESM.tiff]
